# Selection and Characterization of Non‐*Saccharomyces* Yeast Strains for Potential Use in Arabica and Conilon Coffee Fermentations

**DOI:** 10.1111/1750-3841.70431

**Published:** 2025-07-25

**Authors:** Pâmela Mynsen Machado Martins, Iaramarum de Jesus Falcão, Nádia Nara Batista, Patricia Campos Bernardes, Rosane Freitas Schwan

**Affiliations:** ^1^ Food Science Department Federal University of Lavras Lavras Minas Gerais Brazil; ^2^ Biology Department Federal University of Lavras Lavras Minas Gerais Brazil; ^3^ Federal University of Espírito Santo Alegre Espírito Santo Brazil

## Abstract

Customized fermentation approaches are necessary due to the differences in the characteristics of *Coffea arabica* (Arabica) and *Coffea canephora* (Conilon) coffees. Therefore, this work aimed to select and characterize new non‐*Saccharomyces* yeast strains with potential use as starter cultures for Arabica and Conilon coffee fermentation, using a coffee peel and pulp medium (CPM). Fifty‐six yeast strains were assessed for pectinolytic activity, and 34 were selected for further evaluation in CPM. Their performance was assessed based on population growth, °Brix, pH, and production of organic acids. Seventeen strains showed significant growth in Arabica CPM and eight in Conilon CPM, ranging from 10.96% to 18.38%. Nine yeast strains showed higher organic acid production in Arabica and five in Conilon, ranging from 0.826 to 1.336 g/L. Volatile compound production was also evaluated, with 44 compounds detected in Arabica and 61 in Conilon fermentations, showing strain‐dependent profiles. *Meyerozyma guilliermondii* CCMA1737, *Meyerozyma caribbica* (CCMA1993, CCMA1617, CCMA1992, CCMA1950, and CCMA1735), *Pichia kluyveri* CCMA1658, *Cystofilobasidium ferigula* CCMA1647, and *Hanseniaspora uvarum* CCMA1944 were considered the most promising candidates for fermentations with Arabica coffee. *Hanseniaspora uvarum* (CCMA1895 and CCMA1944), *Rhodotorula mucilaginosa* CCMA1663, *P. kluyveri* CCMA1652, and *M. caribbica* CCMA1950 were considered the most promising candidates for Conilon fermentation. For the first time*, H. uvarum* and *R. mucilaginosa* were selected as potential starter cultures for Conilon coffee fermentation. Future studies should evaluate the suitability of these selected yeasts as starter cultures in coffee fermentation.

## 1 Introduction

The main coffee species grown in the world are *Coffea arabica* and *Coffea canephora* (ICO 2024). Among its notable differences, the sensory profile stands out. *C. arabica* coffees have a more delicate, sweet flavor and excellent acidity. *C. canephora* coffees are fuller‐bodied, with lower acidity and more significant bitterness (Jeszka 2022). However, due to advances on and off the field, Brazilian Canephora coffees also attracted attention in the specialty coffee category (Baqueta et al. 2023).

In post‐harvest processing, the fermentation process contributes to the production of coffees with diverse sensory profiles, as it enables the formation of primary and secondary metabolites by microorganisms (Mahingsapun et al. 2022; Bravim et al. 2023; Elhalis et al. 2023; Jimenez et al. 2023). Fermentation aims to reduce the drying time of the beans, enhance flavor precursors, and increase the quality of the beverage. In this process, many biochemical reactions occur inside and outside the beans, and microbial activity in coffee fruits' skin, pulp, and mucilage is intense (Schwan et al. 2022).

Thus, microorganisms perform essential functions in coffee fermentation. The first involves the production of enzymes, known as pectinases, to break down pectin, a primary component of the mucilage layer in coffee fruits. The main enzymes involved are polygalacturonase (PG), pectin lyase (PL), and pectin methylesterase (PME). PG catalyzes the hydrolysis of α‐1,4 glycosidic bonds in polygalacturonic acid. PL catalyzes the degradation of pectin by trans‐elimination, releasing unsaturated galacturonic acids. Finally, PME is responsible for de‐esterifying the pectin's methoxyl group, forming pectic acid and methanol (Agate and Bhat 1966; Silva et al. 2013).

Furthermore, during the fermentation process, microorganisms produce metabolites that can migrate into the beans, contributing exotic characteristics to the flavor and aroma of the beverage (Hadj Salem et al. 2020). These metabolites include volatile and non‐volatile compounds such as organic acids, esters, aldehydes, ketones, and higher alcohols (Prakash et al. 2022; Shankar et al. 2022; Cassimiro et al. 2023).

To transform the fermentation process into a controlled one, developing starter cultures with these properties is crucial for achieving efficient fermentation and producing high‐quality, standardized coffees (Elhalis et al. 2021a). A starter culture comprises a selection of microorganisms prepared to initiate and accelerate fermentation, alter the substrate, increase process efficiency, induce favorable sensory changes, and ensure uniform product development (Holzapfel 1997; Gutiérrez‐Ríos et al. 2022). Among microorganisms, yeasts are an essential group as they play a crucial role in the degradation of mucilage and the production of desirable metabolites (Elhalis et al. 2023; Jimenez et al. 2023).

Several studies have demonstrated that the use of specific yeast strains under controlled fermentation conditions can yield unique and desirable flavor characteristics, thereby enhancing the value of specialty coffees (Da Mota et al. 2020; Silva et al., 2024). As reviewed by Pereira et al. (2024), the use of yeasts in post‐harvest fermentation has transformed the coffee industry by enabling the creation of novel and differentiated sensory profiles, thereby helping producers meet the demands of increasingly discerning markets.

Therefore, as the coffee industry continually seeks innovative methods to increase product quality, the search for new strains of yeast becomes essential. However, customized fermentation approaches are required due to the differences in the characteristics of Arabica and Conilon coffees. Therefore, this work aimed to select new yeast strains that could be used as starter cultures during the fermentation of Arabica and Conilon coffees and evaluate their performance in coffee peel and pulp medium (CPM).

## 2 Materials and Methods

### 2.1 Microorganisms

The yeast strains (56) used in this work were obtained from the Agricultural Microbiology Culture Collection (CCMA, Universidade Federal de Lavras, Lavras, MG). *Cystofilobasidium ferigula*, *Rhodotorula mucilaginosa*, *Debaryomyces hansenii*, *Wickerhamomyces anomalus*, *Hanseniaspora uvarum*, *Hanseniaspora opuntiae*, *Pichia kluyveri*, *Pichia fermentans*, *Meyerozyma guilliermondii*, *and Meyerozyma caribbica*, were previously isolated from fruits of *C. arabica* and *C. canephora* (Table S1) (Evangelista et al. 2015; Martins et al. 2020; Pereira et al. 2021; Pereira et al. 2022; Ribeiro et al. 2018). The isolates (1 mL) stored at −80°C in YEPG containing 20% glycerol (w/w) were reactivated and grown in YEPG medium (in g L^−1^: yeast extract 10 [Himedia], glucose 20 [Dynamics], peptone 20 [Himedia]).

### 2.2 Pectinase Activity of Yeasts

The yeast's pectinolytic activity was determined by the methods described by Schwan et al. (1997) and Silva et al. (2013) with modifications. The yeasts were grown on plates containing MP5 mineral medium (0.5% glucose, 0.5% polygalacturonic acid, 0.6% KH_2_PO_4_, 0.1% yeast extract, 0.2% (NH4) SO_4_, 1.5% agar, and 0.1 mL of the solutions: FeSO_4_ 0.0001%, MgSO_4_ 0.02%, CaCl_2_ 0.0002%, H_3_BO_3_ 0.0002%, 0.0002% MnSO_4_ 0.0014% ZnSO_4_·7H_2_O, and 0.001% CuSO_4_·5H_2_O) for determination of the PG enzyme activity. The determination of PL enzyme activity was carried out in an MP7 medium. This medium had the same constitution as MP5, except for substituting 0.5% polygalacturonic acid for 0.5% citrus pectin. A 1% solution of cetyl trimethyl ammonium bromide (CTAB) was used as the revealing solution for the enzymatic assay, being considered positive when a clear halo formed around the colony. The pectinase activity of yeasts was carried out in triplicate.

### 2.3 Yeast Growth Evaluation in Coffee‐Based Medium

The yeasts selected in the enzymatic test (34) were inoculated into a culture medium containing CPM as described by Martins et al. (2022), with modifications. *Coffea arabica* and *C. canephora* were used separately with all yeasts. The CPM medium was prepared with 20% coffee peel and pulp and 1.5% glucose. First, the CPM was mixed with distilled water, homogenized in a blender for 5 min, and filtered. Then glucose was added, and the pH was adjusted to 5.5 with HCl (0.1 M). The CPM medium was sterilized at 121°C for 15 min.

Yeast was grown in YEPG medium to a concentration of 10⁸ cells/mL. The yeast cells were centrifuged and resuspended in a CPM medium. Thus, 10 mL of inoculum (10⁸ CFU/mL) was transferred separately to flasks containing 90 mL of CPM medium and kept at 28°C for 48 h. Microorganisms were quantified with serial dilution, followed by plating in micro‐drops in YEPG culture medium at pH 3.5. The pH (indicator strips—Sulpelco, Germany) and concentration of soluble solids (°Brix—refractometer, Lorben, Brazil) were measured at the beginning (0 h) and at the end of fermentation (48 h). Samples (0, 24, and 48 h) were collected and stored at −20°C for subsequent analyses of organic acids and volatile compounds. The fermentations were carried out in triplicate.

### 2.4 Determination of Organic Acids

Organic acids were evaluated at the beginning (0 h) and at the end of fermentation (48 h). The samples were centrifuged (12,745 × *g* at 4°C for 10 min), and a perchloric acid solution was used to adjust the pH of the supernatant to 2.11. Then, the supernatant was centrifuged under the same conditions, filtered through a 0.22‐µm cellulose acetate membrane, and stored at −18°C until analysis (Martins et al. 2022). The extracts were evaluated by high‐performance liquid chromatography (LC‐10Ai, Shimadzu Corp., Japan), according to Da Mota et al. (2020). Compound quantification was performed using calibration curves constructed with different concentrations of standard compounds (malic and citric acids were purchased from Merck [Darmstadt, Germany], and lactic, acetic, and succinic acids were purchased from Sigma‐Aldrich [Saint Louis, MO, United States]). All analyses were performed in triplicate. In Table 3, the column “Organic acids” refers to the final concentration (g/L) of each compound after 48 h of fermentation, while the column “Organic acids production” represents the net production by each yeast strain, calculated as the difference between the concentrations at 48 h and the initial values in the CPM medium at 0 h.

### 2.5 Determination of Volatile Compounds

For volatile compound analysis, a subset of strains was selected based on their high performance in growth and organic acid production. A total of nine strains were chosen for Arabica and five for Conilon, reflecting those with the most promising profiles for potential application in fermentation. Volatile compounds were extracted in the beginning (0 h) and at the end of fermentation (48 h) using a manual headspace solid‐phase microextraction (HS‐SPME) procedure and a 50/30 µm divinylbenzene/carboxen/polydimethylsiloxane SPME fiber (Supelco Co., Bellefonte, PA, USA) (Evangelista et al. 2015). The samples were placed in a 15‐mL hermetically sealed vial. The samples were heated for 15 min at 60°C to reach headspace equilibrium. The SPME fiber was then exposed to the samples for 30 min at the same temperature to extract them. Then, the injections were performed by fiber exposition for 2 min. The compounds were analyzed using a Shimadzu QP2010 gas chromatograph (GC) equipped with a mass spectrometer (MS) and a Carbo‐Wax 20 M silica capillary column (30 m × 0.25 mm × 0.25 mm). The oven temperature was adjusted to 60°C for 5 min, followed by a heating rate of 10°C/min until it reached 230°C, and then maintained at this temperature for 15 min. The carrier gas (He) was kept at a flow rate of 1.9 mL/min (Martinez, Rabelo, et al. 2021). Volatile compounds were identified using GC‐MS software (version 2.6), and the mass spectra of each peak were evaluated in the NIST11 database. Furthermore, a series of alkanes (C10–C40) was used to calculate each compound's retention index (RI) and compare it with RI values from the literature. All samples were analyzed in triplicate.

### 2.6 Statistical Analysis

All experiments were conducted using a completely randomized design (CRD). Two independent experiments were performed using CPM prepared from *C. arabica* and *C. canephora*, respectively. For each experiment, 34 yeast strains were evaluated, and each strain was tested in three biological replicates (separate fermentations), resulting in a total of 102 fermentations per coffee type. Analytical measurements, including microbial population (log CFU/mL), pH, °Brix, and organic acid concentrations, were performed on each biological replicate. The data, including the total of chemical classes, were analyzed using analysis of variance (ANOVA), considering yeast strain as the main factor. Means were compared using the Scott–Knott test at a significance level of 5% (*p* ≤ 0.05), as determined by Sisvar 5.6 software (Ferreira 2014). No interaction terms were included in the model since each strain was evaluated independently. For volatile compound analysis, the production of each chemical class was estimated by calculating the total variation in peak area for each class between 0 h and 48 h. Only classes that showed a positive variation were considered in the analysis. A descriptive approach was used to compare the profiles, and a heatmap was generated using Heatmapper (Babicki et al. 2016) to visualize the distribution of volatile classes among the selected strains.

## 3 Results and Discussion

Pectinolytic enzymes are among the most widely produced enzymes by microorganisms and plants (Whitaker 1990). They are involved in the breakdown and modification of pectins, as well as the release of compounds during coffee fermentation (Patidar et al. 2018). Complete degradation of mucilage is achieved through the presence of microbial pectinases, which are mainly produced by yeast (Elhalis et al. 2023). This study evaluated the pectinolytic activity of 56 yeast strains, and 34 strains were selected. These strains showed positive results for PG and PL enzyme activity (Table 1) and were used in fermentation in a CPM medium. The enzymatic capacity of the selected isolates to hydrolyze the components present in coffee mucilage is a crucial indicator of their suitability as a starter culture (Elhalis et al. 2021a).

**TABLE 1:** Polygalacturonase (PG) and pectin lyase (PL) activity and selection of yeast strains isolated from Arabica and Canephora coffee fruits.

Yeast strain	MP5 medium	MP7 medium	Selected yeast
*Cystofilobasidium ferigula*			
CCMA1619	++	++	Yes
CCMA1621	—	—	
CCMA1623	—	—	
CCMA1647	++	++	Yes
CCMA1636	—	—	
CCMA1649	—	—	
CCMA1651	++	++	Yes
CCMA1654	++	++	Yes
CCMA1665	—	—	
CCMA1631	++	++	Yes
*Debaryomyces hansenii*			
CCMA0468	—	—	
*Hanseniaspora opuntiae* Čadež			
CCMA1942	—	—	
CCMA1733	++	++	Yes
*Hanseniaspora uvarum*			
CCMA1639	—	—	
CCMA1892	++	++	Yes
CCMA1893	—	—	
CCMA1895	++	++	Yes
CCMA1944	++	++	Yes
CCMA1996	—	—	
CCMA 2016	—	—	
*Meyerozyma caribbica*			
CCMA1615	—	—	
CCMA1617	++	++	Yes
CCMA1624	++	++	Yes
CCMA1634	++	++	Yes
CCMA1635	—	—	
CCMA1949	—	—	
CCMA1950	++	++	Yes
CCMA1951	++	++	Yes
CCMA1952	++	++	Yes
CCMA1953	—	—	
CCMA1954	++	++	Yes
CCMA1992	++	++	Yes
CCMA1993	++	++	Yes
CCMA1995	++	++	Yes
CCMA1734	++	++	Yes
CCMA1735	++	++	Yes
CCMA1736	—	—	
*Meyerozyma guilliermondii*			
CCMA1616	—	—	
CCMA1653	++	++	Yes
CCMA1737	++	++	Yes
CCMA1738	++	++	Yes
CCMA1739	++	++	Yes
CCMA1740	—	—	
*Pichia fermentans*			
CCMA0465	—	—	
CCMA0466	—	—	
*Pichia kluyveri*			
CCMA1652	++	++	Yes
CCMA1658	++	++	Yes
*Rhodotorula mucilaginosa*			
CCMA1622	—	—	
CCMA1637	++	++	Yes
CCMA1646	—	—	
CCMA1662	++	++	Yes
CCMA1663	++	++	Yes
*Wickerhamomyces anomalus*			
CCMA1640	++	++	Yes
CCMA1650	++	++	Yes
CCMA1659	++	++	Yes
CCMA1660	++	++	Yes

### 3.1 Behavior of Selected Yeasts in Coffee Medium

*Saccharomyces cerevisiae* and *Torulaspora delbrueckii* strains have been widely investigated in coffee fermentations due to their well‐established fermentative efficiency and metabolic versatility (Martins et al. 2022; Jimenez et al. 2023; Wang et al. 2020). However, the exploration of non‐*Saccharomyces* yeasts offers an opportunity to further diversify the sensory profiles of coffee. Yeast selection plays a strategic role in modulating the biochemical transformations during fermentation, enabling the production of desirable volatile and non‐volatile compounds (Elhalis et al. 2021b; Pereira et al. 2024; Bravim et al. 2023). Thus, the investigation and characterization of novel yeast candidates make this work highly relevant from both scientific and technological perspectives.

Firstly, the behavior of the selected yeast during fermentation in Arabica and Conilon coffee media was evaluated (Table 2). Seventeen strains showed significant growth in CPM medium (*C. ferigula* (CCMA1619, CCMA1647, and CCMA1654), *W. anomalus* (CCMA1660 and CCMA1640), *H. uvarum* (CCMA1944), *R. mucilaginosa* (CCMA1622), *P. kluyveri* (CCMA1652 and CCMA1658), *M. caribbica* (CCMA1952, CCMA1951, CCMA1954, CCMA1950, CCMA1993, and CCMA1735), and *M. guilliermondii* (CCMA1737 and CCMA1738)), with population increases of 10.97%–17.18%.

**TABLE 2:** Yeast population data, °Brix, and pH during fermentations in coffee peel and pulp medium (CPM) of Arabica and Canephora coffees.

Yeast strains	*Coffea arabica*					*Coffea canephora*				
	Yeasts population (log cells/mL)		Population growth (%)	End of fermentation		Yeasts population (log cells/mL)		Population growth (%)	End of fermentation	
	Time (h)			°Brix	pH	Time (h)			°Brix	pH
	0	48				0	48			
***Cystofilobasidium ferigula***										
CCMA 1631	6.89	7.55	9.58b	4.00i	4.00a	6.72	7.85	16.81b	2.60c	4.00a	
CCMA 1619	6.87	8.05	17.18a	4.50L	5.00c	6.74	7.98	18.40b	4.20k	5.00c	
CCMA 1647	6.84	7.81	14.18a	2.40c	4.00a	7.02	7.54	7.41d	2.00a	4.50b	
CCMA 1651	6.76	7.41	9.61b	4.00i	4.50b	7.19	7.69	6.95d	4.00j	4.50b	
CCMA 1654	7.02	7.79	10.97a	4.00i	4.50b	6.99	7.87	12.59c	4.00j	4.50b	
*Wickerhamomyces anomalus*											
CCMA 1660	7.05	7.90	12.06a	2.70d	4.50b	6.80	7.93	16.62b	2.80d	4.00a	
CCMA 1659	7.24	7.90	9.12b	3.80f	4.50b	7.09	7.93	11.85c	2.80d	4.00a	
CCMA 1650	6.90	7.57	9.71b	2.20b	4.00a	6.79	7.92	16.64b	3.00e	4.50b	
CCMA 1640	6.74	7.81	15.88a	2.50c	5.00c	7.21	8.02	11.23c	2.00a	4.00a	
***Hanseniaspora uvarum***											
CCMA 1944	7.25	8.12	12.00a	2.00a	4.50b	7.60	8.27	8.82d	2.80d	4.50b	
CCMA 1895	7.67	7.91	3.13c	3.00f	4.00a	7.20	8.27	14.86c	2.40b	4.00a	
CCMA 1892	7.37	8.04	9.09b	3.00f	4.00a	7.68	8.29	7.94d	2.80d	4.50b	
***Hanseniaspora opuntiae cadez***											
CCMA 1733	7.36	7.89	7.20b	2.80d	4.50b	7.40	8.06	8.92d	2.80d	5.00c	
***Rhodotorula mucilaginosa***											
CCMA 1622	6.94	7.90	13.83a	4.50l	5.00c	7.20	8.03	11.53c	3.80h	4.50b	
CCMA 1663	7.05	7.78	10.35b	4.00i	4.50b	6.85	7.65	11.68c	4.00j	4.50b	
CCMA 1637	7.75	7.88	1.68c	4.00i	5.00c	6.39	7.79	21.91a	2.80d	4.50b	
***Pichia kluyveri***											
CCMA 1652	6.92	8.04	16.18a	3.70g	5.00c	6.98	7.78	11.46c	2.80d	5.00c	
CCMA 1658	7.08	8.06	13.84a	2.80d	4.00a	7.00	7.95	13.57c	2.80d	4.00a	
***Meyerozyma caribbica***											
CCMA 1617	7.10	7.60	7.04b	5.00m	4.50b	6.99	8.02	14.74c	4.20k	4.50b	
CCMA 1624	7.08	7.67	8.33b	4.50l	4.00a	7.15	7.83	9.51d	4.20k	4.50b	
CCMA 1634	6.82	7.48	9.67b	4.50l	4.00a	7.25	7.78	7.31d	2.60c	4.50b	
CCMA 1952	7.07	7.92	12.02a	3.90h	5.00c	7.06	8.16	15.58b	3.60f	4.50b	
CCMA 1951	6.95	7.73	11.22a	4.00i	4.50b	7.10	7.96	12.11c	3.00e	4.50b	
CCMA 1954	7.19	8.18	13.80a	2.40c	4.50b	6.85	7.74	12.99c	4.00j	5.00c	
CCMA 1950	7.07	8.20	15.98a	2.80d	4.50b	7.09	7.19	1.41e	2.60c	4.50b	
CCMA 1995	7.17	7.79	8.65b	4.20k	5.00c	7.27	8.23	13.20c	3.70g	5.00c	
CCMA 1992	7.16	7.84	9.50b	3.80f	5.00c	6.83	7.90	15.67b	4.00 j	4.50b	
CCMA 1993	7.00	8.00	14.29a	2.80d	4.50b	7.10	8.12	14.37c	4.00j	5.00c	
CCMA 1734	7.38	7.84	6.23b	4.50l	4.50b	7.24	7.86	8.56d	4.00j	5.00c	
CCMA 1735	6.90	7.95	15.22a	4.00i	5.00c	6.95	8.10	16.55b	4.00j	4.50b	
***Meyerozyma guilliermondii***											
CCMA 1737	6.96	7.86	12.93a	4.00i	5.00c	7.29	7.79	6.86d	3.80h	4.50b	
CCMA 1738	6.89	7.76	12.63a	4.50L	4.50b	6.94	7.83	12.82c	3.90i	4.50b	
CCMA 1739	7.30	7.82	7.12b	4.10j	4.50b	7.15	8.18	14.41c	3.60f	4.50b	
CCMA 1653	7.08	7.56	6.78b	2.90e	4.50b	7.27	7.75	6.60d	3.80h	4.50b	
**Standard error of the mean**	0.062	0.084	1.606	0.032	0.015	0.080	0.032	1.216	0.015	0.018	

*Note*: Data are presented as mean. a–m for each column, mean values with different letters are significant at *p* ≤ 0.05 by Scott–Knott test.

Each strain contributes uniquely to the coffee fermentation process. The population of these species varies with the chemical alterations established during coffee processing. *Cystofilobasidium ferigula* was the most abundant yeast detected in Arabica coffee fruit by the next‐generation sequencing technique, mainly in coffee grown at 800 and 1200 m. Other species, such as *M. caribbica* and *W. anomalus*, were more abundant at 1000–1400 and 1200 m, respectively (Martinez, Simão, et al. 2021). *Hanseniaspora uvarum* also proved to be a dominant yeast in the first hours of fermentation in a synthetic coffee pulp extract medium. Elhalis et al. (2021a) detected a population of 4.7 log CFU/mL, reaching a maximum population (11.6 log CFU/mL) after 36 h of fermentation.

*Rhodotorula mucilaginosa* population (5 log CFU/g) remained constant in Bourbon variety coffees grown at 700–800 m (Vilela et al. 2010). *Pichia kluyveri*, described in other geographical areas and processes, reached 7.53 log CFU/g during fermentation in Tanzanian coffees (Masoud et al. 2004). Studies have shown that during coffee fermentation, *R. mucilaginosa* and *P. kluyveri* may inhibit ochratoxin A production (Souza et al. 2017). This finding suggests that using specific strains that inhibit ochratoxin A production may be a promising alternative for the coffee fermentation process.

Pereira et al. (2021) evaluated the microbial diversity in Conilon coffee in four different environments. *Candida*, *Meyerozyma*, *Hanseniaspora*, and *Pichia* were the primary yeast genera. Notably, *M. caribbica* and *M. guilliermondii* were present in all environments studied, while *P. kluyveri* was only found in coffee cultivated at 600 m.

In the present study, the population growth of certain yeast strains stood out in the *C. canephora* coffee medium. For instance, in the CPM medium fermented with *R. mucilaginosa* CCMA1637, there was a substantial increase of 21.91%. Similarly, the strains *C. ferigula* (CCMA1631 and CCMA1619), *W. anomalus* (CCMA1660 and CCMA1650), and *M. caribbica* (CCMA1952, CCMA1992, and CCMA1735) achieved growth between 15.58% and 18.40%, demonstrating their robustness and likely potential to be used in the Conilon fermentation.

Yeast growth involves depleting the substrate and producing organic acids, which can be verified by measuring the reduction in soluble solid concentration and pH. However, the ability to decrease the °Brix and pH varies among species and within strains (Haile and Kang 2019). The Arabica coffee medium presented 5 °Brix and pH 5. After 48 h of fermentation, lower concentrations of soluble solids (2 °Brix) were obtained for fermentation with *H. uvarum* CCMA1944. The lower pH (2) was obtained for fermentation with *C. ferigula* (CCMA1631 and CCMA1647), *W. anomalus* (CCMA1650), *H. uvarum* (CCMA1895 and CCMA1892), *P. kluyveri* (CCMA1658), and *M. caribbica* (CCMA1624 and CCMA1634). Regarding Conilon coffee, the medium presented 5.5 °Brix and pH 5. After 48 h, fermentations with *C. ferigula* CCMA1647 and *W. anomalus* (CCMA1640) obtained the lowest concentration of soluble solids (2 °Brix). Fermentations with *C. ferigula* (CCMA1631), *W. anomalus* (CCMA1660, CCMA1659, and CCMA1640), *H. uvarum* (CCMA1895), and *P. kluyveri* (CCMA1658) obtained the lowest pH of 4.

### 3.2 Organic Acids Production

The volatile and non‐volatile compounds in the coffee beverage directly affect sensory quality, making their evaluation extremely important, as understanding that the yeast strain plays a crucial role in influencing the presence and concentration of these compounds during fermentation (Prakash et al. 2022; Jimenez et al. 2023; Elhalis et al. 2021b). Different organic acids have already been detected in coffee. These acids contribute to the beverage's acidity, perception of sweetness, and refreshing and fruity flavors, which are essential for the coffee's quality (Farah and de Lima 2019). Thus, the total production of organic acids during the fermentation process was evaluated to select the potential strains for the fermentation of Arabica and Conilon coffee (Table 3).

**TABLE 3:** Organic acids detected during fermentation by high‐performance liquid chromatography (HPLC).

Fermentation time (h)	Treatments	*Coffea arabica*					*Coffea canephora*				
		Organic acids (g/L)				Organic acids production (g/L)^b^	Organic acids (g/L)				
		Citric	Malic	Succinic			Citric	Malic	Succinic		Organic acids production (g/L)^b^
0	CPM medium^a^	1.94	1.11	0.66			2.09	0.97	0.63		
48	***Cystofilobasidium ferigula***										
	CCMA 1631	2.23	0.18	0.79		0.411d	1.63	nd	1.27		0.643b
	CCMA 1619	2.17	nd	0.51		0.221d	1.69	0.91	1.08		0.449c
	CCMA 1647	2.57	nd	0.99		0.953b	1.72	nd	1.04		0.411c
	CCMA 1651	1.54	0.89	0.43		0.000e	1.41	0.41	0.26		0.000d
	CCMA 1654	1.92	1.31	0.67		0.217d	2.00	0.59	1.02		0.401c
	*Wickerhamomyces anomalus*										
	CCMA 1660	2.19	nd	0.66		0.268d	1.78	0.09	0.43		0.000d
	CCMA 1659	2.59	nd	0.68		0.673c	1.80	0.06	0.42		0.000d
	CCMA 1650	1.76	nd	0.58		0.000e	1.69	nd	0.35		0.000d
	CCMA 1640	2.16	nd	0.75		0.303d	1.94	nd	1.34		0.713b
	***Hanseniaspora uvarum***										
	CCMA 1944	2.88	0.13	0.69		0.989b	2.61	nd	1.45		1.336a
	CCMA 1895	1.85	nd	0.64		0.008e	1.86	nd	1.52		0.889a
	CCMA 1892	2.03	nd	0.60		0.089e	2.00	nd	1.39		0.760b
	***Hanseniaspora opuntiae cadez***										
	CCMA 1733	2.16	nd	0.99		0.545c	1.83	nd	0.80		0.172d
	***Rhodotorula mucilaginosa***										
	CCMA 1622	2.41	1.31	0.70		0.720c	1.88	0.64	1.22		0.588b
	CCMA 1663	1.85	1.10	0.48		0.029e	2.39	0.14	1.31		0.979a
	CCMA 1637	1.93	1.16	0.55		0.228d	1.80	nd	1.44		0.818b
	***Pichia kluyveri***										
	CCMA 1652	2.28	nd	0.93		0.606c	2.19	nd	1.50		1.013a
	CCMA 1658	2.56	nd	0.997		0.930b	2.46	0.02	0.53		0.363c
	***Meyerozyma caribbica***										
	CCMA 1617	2.39	1.49	0.64		0.826b	1.70	0.52	1.06		0.438c
	CCMA 1624	2.43	1.26	0.67		0.648c	1.36	0.51	0.27		0.000d
	CCMA 1634	1.51	0.91	0.37		0.000e	2.43	nd	1.05		0.760b
	CCMA 1952	2.18	1.26	0.74		0.456d	2.28	0.55	0.64		0.209d
	CCMA 1951	1.87	0.83	0.68		0.103e	2.01	nd	1.17		0.667b
	CCMA 1954	2.07	0.27	0.51		0.131e	1.84	0.54	0.98		0.358c
	CCMA 1950	2.57	nd	0.87		0.835b	1.91	nd	1.72		1.105a
	CCMA 1995	2.21	1.21	0.53		0.360d	1.73	0.52	1.09		0.462c
	CCMA 1992	2.47	1.37	0.71		0.832b	2.03	0.73	1.19		0.559b
	CCMA 1993	3.21	nd	0.82		1.421a	1.60	0.45	0.37		0.000d
	CCMA 1734	2.13	1.11	0.56		0.216d	2.28	0.80	0.63		0.219d
	CCMA 1735	2.30	1.53	0.74		0.857b	1.82	0.49	1.17		0.545b
	***Meyerozyma guilliermondii***										
	CCMA 1737	3.90	1.88	0.86		1.277a	1.88	0.11	1.10		0.477c
	CCMA 1738	1.66	0.69	0.40		0.000e	1.99	0.63	1.14		0.527b
	CCMA 1739	2.39	1.18	0.54		0.510c	2.18	0.05	1.28		0.841b
	CCMA 1653	2.13	1.48	0.48		0.547c	1.85	0.48	1.19		0.559b
**Standard error of the mean**						0.089					0.109
										

*Note*: Data are presented as mean. Letters a–e for each column, mean values with different letters are significant at *p* ≤ 0.05 by Scott–Knott test.

Abbreviation: nd = not detected.

^a^ Coffee peel and pulp medium.

^b^ Organic acid production was calculated as the difference between the concentration measured at 48 h and at the start of fermentation (0 h).

In Arabica coffee, the strains that showed the highest production of organic acids during fermentation in CPM medium were *M. caribbica* CCMA1993 (1.421 g/L) and *M. guilliermondii* CCMA1737 (1.277 g/L). Furthermore, *C. ferigula* CCMA1647, *H. uvarum* CCMA1944, *P. kluyveri* CCMA1658, and *M. caribbica* (CCMA1617, CCMA1950, CCMA1992, and CCMA1735) showed significant production, ranging from 0.826 to 0.989 g/L. Silva et al. (2013) evaluated the production of organic acids by *S. cerevisiae* and non‐*Saccharomyces*. Most non‐*Saccharomyces* strains showed better results for succinic acid production.

In Conilon coffee, the strains that produced the highest concentration of organic acids during fermentation in the CPM medium were *H. uvarum* (CCMA1944 and CCMA1895), *R. mucilaginosa* (CCMA1663), *P. kluyveri* (CCMA1652), and *M. caribbica* (CCMA1950), with concentrations ranging from 0.889 to 1.336 g/L.

The formation of succinic, malic, and citric acids depends on the yeast strain's genetic background. Furthermore, other factors can influence its formation, including aeration conditions, temperature, the chemical composition of the growth medium, and pH (Ferreira and Mendes‐Faia 2020). Free succinic acid imparts an unusually salty and bitter taste regarding sensory perception. On the other hand, malic acid has a mild flavor, is less acidic than citric acid, and is more persistent. Both contribute fruity notes, such as green apple and fresh acidity (Farah and de Lima 2019). In addition to contributing to the beverage's acidity, organic acids produced by yeast during fermentation may act as substrates in the biosynthesis of esters. These important aromatic compounds contribute to the fruity and floral sensorial profile of fermented beverages (Ferreira and Mendes‐Faia 2020). The acids in green beans may also affect the formation of critical volatile compounds during coffee roasting, such as pyrazines and furans (Yu and Zhang 2010).

Overall, the strains *H. uvarum* CCMA1944 and *M. caribbica* CCMA1950, isolated from Arabica coffee fruits, were found to be capable of producing significant concentrations of organic acids in both *C. arabica* and *C. canephora* fermentations. These promising results could lead to potential applications in the coffee industry, contributing to the improvement of coffee fermentation processes.

### 3.3 Volatile Compounds Production

Volatile compounds were analyzed in the media of fermented coffees, totaling nine fermentations of Arabica coffee (Table S2) and five fermentations of Conilon coffee (Table S3). Forty‐four compounds were detected in the medium fermented with Arabica coffee, belonging to the classes of acids (8), alcohols (12), aldehydes (3), ketones (3), esters (14), and phenols (4). On the other hand, in the medium fermented with Conilon coffee, sixty‐one compounds were found, belonging to the classes of acids (7), alkanes (4), alcohols (14), aldehydes (2), ketones (3), esters (27), and phenols (4).

**FIGURE 1:**
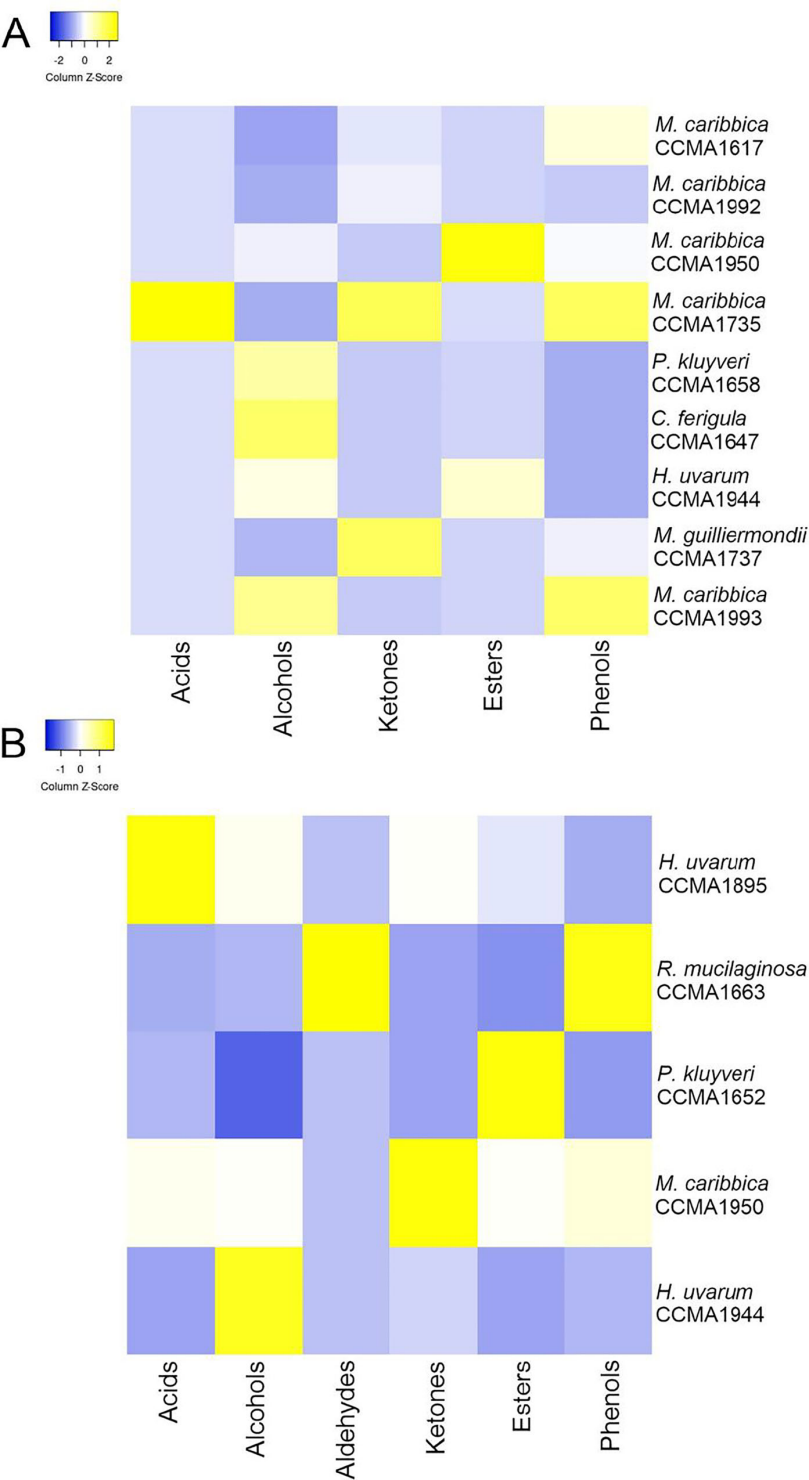
Heatmap of the classes of volatile compounds produced in the coffee peel and pulp medium (CPM) using *Coffea arabica* (A) and *Coffea canephora* (B).

The production of volatile compounds during fermentation was calculated using the difference in the area of volatile compounds at the end (48 h) and at the beginning (0 h) of fermentation (Figure 1).

Some volatile compounds were detected by all yeast strains, namely, 1‐octanol; E,E‐2,13‐octadecadien‐1‐ol; cyclopentanetridecanoic acid, methyl ester; and benzenedicarboxylic acid, bis(2‐methylpropyl) ester, present in *C. arabica*, and octanoic acid; 1‐octanol, 2‐butyl‐; and sulfurous acid, 2‐propyl tetradecyl ester, present in *C. canephora*.

Volatile compounds originating from microbial metabolism differed between strains. In this sense, we can highlight some volatiles produced exclusively by each strain. In *C. arabica*, nonanoic acid (CCMA1735), 1‐butanol, 3‐methyl‐ (CCMA1737), and 4‐hydroxy‐3‐methylacetophenone (CCMA1944). In *C. canephora*, 6‐octadecenoic acid, methyl ester, (Z)‐ (CCMA1663).

Esters, ketones, and aldehydes are the main classes of aromatic compounds (López‐Galilea et al. 2006). In Arabica coffee, *M. caribbica* CCMA1950, followed by *H. uvarum* CCMA1944, showed higher production of esters such as dodecanoic acid, ethyl ester (2951926 area), and tetradecanoic acid, ethyl ester (169067 area). Among those that significantly produced high concentrations of ketones, *M. caribbica* CCMA1735 showed the highest production of ketones, such as 2‐pentadecanone, 6, 10, 14‐trimethyl‐ (11915 area).

In Conilon coffee, each strain used produced at least one chemical class better. *Pichia kluyveri *CCMA1652 was better at producing esters, such as Acetic acid, 2‐phenylethyl ester (20763282 area). *Meyerozyma caribbica* CCMA1950 was better at producing ketones, such as 5,9‐Undecadien‐2‐one, 6,10‐dimethyl‐, (Z) (1022528 area). *Rhodotorula mucilaginosa *CCMA1663 was better at producing aldehydes, such as Benzaldehyde 2,4‐dimethyl‐ (103851 area).

Microbial metabolism exhibits distinct variations in its phenotypes and metabolites, which modify metabolic pathways and yield interesting compounds (Yi and Alper 2022). Non‐*Saccharomyces* yeasts have demonstrated considerable ester production, resulting in greater flavor complexity and variability in fermented foods (Malićanin et al. 2022). Some yeasts, such as *Pichia*, have stood out in producing acetic acid, 2‐phenylethyl ester, the principal aromatic component in the food and fragrance industries due to its flowery flavor (Chung et al. 2000). Wang et al. (2020) obtained a 2.4‐fold increase in the production of acetic acid, 2‐phenylethyl ester, after the fermentation of arabica coffee inoculated with *P. kluyveri*. Strains of *H. uvarum* have been evaluated as starter cultures for the coffee fermentation process. Elhalis et al. (2021a) detected significant ester production in a synthetic coffee pulp extract medium. Arabica coffee beans produced 2–3 times higher concentrations of metabolites such as glycerol, alcohols, aldehydes, esters, and organic acids (Elhalis et al. 2021b). In this work, the primary ester produced by *H. uvarum* was dodecanoic acid, ethyl ester, a compound originating from its metabolism (Mestre et al. 2019).

The group of ketones and aldehydes also produces relevant volatile compounds that contribute to the flavor of coffee. The ketone group accounts for 20%–27% of coffee's aromatic components (Dippong et al. 2022). The primary volatile aromatics produced by *M. caribbica* CCMA1735 and *M. guilliermondii* CCMA1737 were 2‐pentadecanone, 6, 10, 14‐trimethyl‐ (floral odor) in *C. arabica*, and *M. caribbica* CCMA1950 produced 5,9‐Undecadien‐2‐one, 6,10‐dimethyl‐, (Z) (floral flavor) in *C. canephora*.

Aldehydes represent about 10% of the total volatiles in green beans, and they have generally shown a positive correlation with important aromatic compounds obtained in inoculated fermentations (Elhalis et al. 2021b). The volatile compound of *R. mucilaginosa* has been little explored in coffee. This yeast is known for its ability to produce carotenoids, and its presence during the fermentation process can effectively inhibit OTA production (Moreira et al. 2018; Souza et al. 2017). This is the first study to select *R. mucilaginosa* and *H. uvarum* as potential starter cultures for the fermentation of Conilon coffee.

In contrast to traditional coffee fermentation strains such as *S. cerevisiae* and *P. fermentans*, which have been widely studied for their pectinolytic activity, stress tolerance, and ester production (Pereira et al. 2024), the strains selected in this study, including *H. uvarum* and *R. mucilaginosa*, demonstrated potential to enhance the aromatic complexity of *C. canephora* fermentations. Notably, these non‐*Saccharomyces* strains exhibited prominent production of esters and ketones, chemical classes associated with fruity and floral notes. Moreover, the use of *H. uvarum* and *R. mucilaginosa* may offer advantages in diversifying flavor profiles without the dominance typically seen in *S. cerevisiae*‐driven fermentations, supporting their suitability as novel starter cultures for Conilon coffee.

## 4 Conclusion

The main findings of this study were as follows: (I) 34 strains showed pectinolytic activity and (II) in *C. arabica*, *M. caribbica* CCMA1950 and *H. uvarum* CCMA1944 showed better growth and ester production. (III) In *C. canephora*, *P. kluyveri *CCMA1652 showed enhanced esters and organic acid production, *M. caribbica* CCMA1950 stood out for ketones and organic acid production, and *R. mucilaginosa* CCMA1663 for aldehyde and organic acid production. (IV) *M. guilliermondii* CCMA1737, *M. caribbica* (CCMA1993, CCMA1617, CCMA1992, CCMA1950, and CCMA1735), *P. kluyveri* CCMA1658, *C. ferigula* CCMA1647, and *H. uvarum* CCMA1944 were considered the most promising candidates for fermentations with Arabica coffee; V) *H. uvarum* (CCMA1895 and CCMA1944), *R. mucilaginosa* CCMA1663, *P. kluyveri* CCMA1652, and *M. caribbica* CCMA1950 were considered the most promising candidates for fermentations with Conilon coffee. Therefore, this study reveals potential new starter cultures for coffee fermentations and, for the first time, identifies *H. uvarum* and *R. mucilaginosa* strains as candidates for *C. canephora* fermentation. Future studies should assess the fermentative performance of these selected yeasts as starter cultures in different *C. arabica* and *C. canephora* cultivars and under varied processing conditions.

## Author Contributions

**Pâmela Mynsen Machado Martins**: conceptualization, investigation, methodology, validation, formal analysis, writing–original draft. **Iaramarum de Jesus Falcão**: investigation, methodology, writing–original draft. **Nádia Nara Batista**: conceptualization, visualization, validation, writing–original draft, supervision. **Patricia Campos Bernardes**: supervision, methodology, visualization, conceptualization. **Rosane Freitas Schwan**: conceptualization, funding acquisition, writing–review and editing, visualization, methodology, project administration, supervision, resources.

## Conflicts of Interest

The authors declare no conflicts of interest.

## Supporting information




**Supporting Material**: jfds70431‐sup‐0001‐SupMat.docx

## References

[jfds70431-bib-0001] Agate, A. D. , and J. V. Bhat . 1966. “Role of Pectinolytic Yeasts in the Degradation of Mucilage Layer of Coffea Robusta Cherries.” Applied Microbiology 14, no. 2: 256–260. http://www.ncbi.nlm.nih.gov/pubmed/5959859. 5959859 10.1128/am.14.2.256-260.1966 PMC546662

[jfds70431-bib-0002] Babicki, S. , D. Arndt , A. Marcu , et al. 2016. “Heatmapper: Web‐Enabled Heat Mapping for All.” Nucleic Acids Research 44, no. W1: W147–53. 10.1093/nar/gkw419. 27190236 PMC4987948

[jfds70431-bib-0003] Baqueta, M. R. , E. A. Alves , P. Valderrama , and J. A. L. Pallone . 2023. “Brazilian Canephora Coffee Evaluation Using NIR Spectroscopy and Discriminant Chemometric Techniques.” Journal of Food Composition and Analysis 116: 105065. 10.1016/j.jfca.2022.105065.

[jfds70431-bib-0004] Bravim, D. G. , T. Mota de Oliveira , D. Kaic Alves do Rosário , et al. 2023. “Inoculation of Yeast and Bacterium in Wet‐Processed *Coffea canephora* .” Food Chemistry 400: 134107. 10.1016/j.foodchem.2022.134107. 36087481

[jfds70431-bib-0005] Cassimiro, D. M. d. J. , N. N. Batista , H. Calixto Fonseca , et al. 2023. “Wet Fermentation of *Coffea canephora* by Lactic Acid Bacteria and Yeasts Using the Self‐Induced Anaerobic Fermentation (SIAF) Method Enhances the Coffee Quality.” Food Microbiology 110: 104161. 10.1016/j.fm.2022.104161. 36462817

[jfds70431-bib-0006] Chung, H. , S. L. Lee , and C. C. Chou . 2000. “Production and Molar Yield of 2‐Phenylethanol by Pichia Fermentans L‐5 as Affected by Some Medium Components.” Journal of Bioscience and Bioengineering 90, no. 2: 142–147. 10.1016/S1389-1723(00)80101-2. 16232833

[jfds70431-bib-0007] Da Mota, M. C. B. , N. N. Batista , M. H. Sances Rabelo , D. E. Ribeiro , F. Meira Borém , and R. F. Schwan . 2020. “Influence of Fermentation Conditions on the Sensorial Quality of Coffee Inoculated With Yeast.” Food Research International 136: 109482. 10.1016/j.foodres.2020.109482. 32846564

[jfds70431-bib-0008] Dippong, T. , M. Dan , M. H. Kovacs , E. D. Kovacs , E. A. Levei , and O. Cadar . 2022. “Analysis of Volatile Compounds, Composition, and Thermal Behavior of Coffee Beans According to Variety and Roasting Intensity.” Foods 11, no. 19: 3146. 10.3390/foods11193146. 36230221 PMC9563260

[jfds70431-bib-0009] Elhalis, H. , J. Cox , D. Frank , and J. Zhao . 2021a. “Microbiological and Biochemical Performances of Six Yeast Species as Potential Starter Cultures for Wet Fermentation of Coffee Beans.” LWT‐Food Science and Technology 137: 110430. 10.1016/j.lwt.2020.110430.

[jfds70431-bib-0010] Elhalis, H. , J. Cox , D. Frank , and J. Zhao . 2021b. “Microbiological and Chemical Characteristics of Wet Coffee Fermentation Inoculated with *Hansinaspora uvarum* and *Pichia kudriavzevii* and Their Impact on Coffee Sensory Quality.” Frontiers in Microbiology 12: 713969. 10.3389/fmicb.2021.713969. 34421873 PMC8371688

[jfds70431-bib-0011] Elhalis, H. , J. Cox , and J. Zhao . 2023. “Yeasts Are Essential for Mucilage Degradation of Coffee Beans During Wet Fermentation.” Yeast 40, no. 9: 425–436. 10.1002/yea.3888. 37464909

[jfds70431-bib-0012] Evangelista, S. R. , M. Gabriela da Cruz Pedroso Miguel , C. Ferreira Silva , A. C. Marques Pinheiro , and R. Freitas Schwan . 2015. “Microbiological Diversity Associated With the Spontaneous Wet Method of Coffee Fermentation.” International Journal of Food Microbiology 210: 102–112. 10.1016/j.ijfoodmicro.2015.06.008. 26119187

[jfds70431-bib-0013] Farah, A. , and Â. Galvan de Lima . 2019. “Organic Acids.” In Coffee: Production, Quality and Chemistry, 517–536. The Royal Society of Chemistry.

[jfds70431-bib-0014] Ferreira, A. M. , and A. Mendes‐Faia . 2020. “The Role of Yeasts and Lactic Acid Bacteria on the Metabolism of Organic Acids During Winemaking.” Foods 9, no. 9: 1231. 10.3390/foods9091231. 32899297 PMC7555314

[jfds70431-bib-0015] Ferreira, D. F. 2014. “Sisvar: a Guide for Its Bootstrap Procedures in Multiple Comparisons.” Ciência e Agrotecnologia 38, no. 2: 109–112. 10.1590/S1413-70542014000200001.

[jfds70431-bib-0016] Gutiérrez‐Ríos, H. G. , M. L. Suárez‐Quiroz , Z. Josué Hernández‐Estrada , et al. 2022. “Yeasts as Producers of Flavor Precursors During Cocoa Bean Fermentation and Their Relevance as Starter Cultures: A Review.” Fermentation 8, no. 7: 331. 10.3390/fermentation8070331.

[jfds70431-bib-0017] Hadj Salem, F. , M. Lebrun , C. Mestres , N. Sieczkowski , R. Boulanger , and A. Collignan . 2020. “Transfer Kinetics of Labeled Aroma Compounds From Liquid Media Into Coffee Beans During Simulated Wet Processing Conditions.” Food Chemistry 322: 126779. 10.1016/j.foodchem.2020.126779. 32305877

[jfds70431-bib-0018] Haile, M. , and W. H. Kang . 2019. “Isolation, Identification, and Characterization of Pectinolytic Yeasts for Starter Culture in Coffee Fermentation.” Microorganisms 7, no. 10: 401. 10.3390/microorganisms7100401. 31569406 PMC6843319

[jfds70431-bib-0019] Holzapfel, W. 1997. “Use of Starter Cultures in Fermentation on a Household Scale.” Food Control 8, no. 5–6: 241–258. 10.1016/S0956-7135(97)00017-0.

[jfds70431-bib-0020] ICO . 2024. “Coffee Market Report—February 2024.” https://icocoffee.org/.

[jfds70431-bib-0021] Jeszka, A. M. 2022. “Current Status of Coffee Production and Global Marketing: Recent Update.” In Coffee Science, 3–13. CRC Press. 10.1201/9781003043133-2.

[jfds70431-bib-0022] Jimenez, E. J. M. , P. M. Machado Martins , A. L. de Oliveira Vilela , et al. 2023. “Influence of Anaerobic Fermentation and Yeast Inoculation on the Viability, Chemical Composition, and Quality of Coffee.” Food Bioscience 51: 102218. 10.1016/j.fbio.2022.102218.

[jfds70431-bib-0023] López‐Galilea, I. , N. Fournier , C. Cid , and E. Guichard . 2006. “Changes in Headspace Volatile Concentrations of Coffee Brews Caused by the Roasting Process and the Brewing Procedure.” Journal of Agricultural and Food Chemistry 54, no. 22: 8560–8566. 10.1021/jf061178t. 17061834

[jfds70431-bib-0024] Mahingsapun, R. , P. Tantayotai , T. Panyachanakul , et al. 2022. “Enhancement of Arabica Coffee Quality With Selected Potential Microbial Starter Culture Under Controlled Fermentation in Wet Process.” Food Bioscience 48: 101819. 10.1016/j.fbio.2022.101819.

[jfds70431-bib-0025] Malićanin, M. , B. Danilović , S. S. Stojanović , et al. 2022. “Pre‐Fermentative Cold Maceration and Native Non‐*Saccharomyces* Yeasts as a Tool to Enhance Aroma and Sensory Attributes of Chardonnay Wine.” Horticulturae 8, no. 3: 212. 10.3390/horticulturae8030212.

[jfds70431-bib-0026] Martinez, S. J. , M. H. S. Rabelo , A. P. Pereira Bressani , M. Caroline Batista Da Mota , F. M. Borém , and R. F. Schwan . 2021. “Novel Stainless Steel Tanks Enhances Coffee Fermentation Quality.” Food Research International 139: 109921. 10.1016/j.foodres.2020.109921. 33509488

[jfds70431-bib-0027] Martinez, S. J. , J. B. P. Simão , V. S. Pylro , and R. F. Schwan . 2021. “The Altitude of Coffee Cultivation Causes Shifts in the Microbial Community Assembly and Biochemical Compounds in Natural Induced Anaerobic Fermentations.” Frontiers in Microbiology 12: 671395. 10.3389/fmicb.2021.671395. 34093490 PMC8172976

[jfds70431-bib-0028] Martins, P. M. M. , N. N. Batista , L. Diniz Santos , D. R. Dias , and R. Freitas Schwan . 2022. “Microencapsulation of Epiphytic Coffee Yeasts by Spray Drying Using Different Wall Materials: Implementation in Coffee Medium.” International Journal of Food Microbiology 379: 109839. 10.1016/j.ijfoodmicro.2022.109839. 35868147

[jfds70431-bib-0029] Martins, P. M. M. , N. N. Batista , M. Gabriela da Cruz Pedrozo Miguel , J. B. Pavesi Simão , J. Ribeiro Soares , and R. Freitas Schwan . 2020. “Coffee Growing Altitude Influences the Microbiota, Chemical Compounds and the Quality of Fermented Coffees.” Food Research International 129: 108872. 10.1016/j.foodres.2019.108872. 32036899

[jfds70431-bib-0030] Masoud, W. , L. Bjørg Cesar , L. Jespersen , and M. Jakobsen . 2004. “Yeast Involved in Fermentation of *Coffea arabica* in East Africa Determined by Genotyping and by Direct Denaturating Gradient Gel Electrophoresis.” Yeast 21, no. 7: 549–556. 10.1002/yea.1124. 15164358

[jfds70431-bib-0031] Mestre, M. V. , Y. P. Maturano , C. Gallardo , et al. 2019. “Impact on Sensory and Aromatic Profile of Low Ethanol Malbec Wines Fermented by Sequential Culture of *Hanseniaspora uvarum* and *Saccharomyces cerevisiae* Native Yeasts.” Fermentation 5, no. 3: 65. 10.3390/fermentation5030065.

[jfds70431-bib-0032] Moreira, M. D. , M. M. Melo , J. M. Coimbra , K. C. dos Reis , R. F. Schwan , and C. F. Silva . 2018. “Solid Coffee Waste as Alternative to Produce Carotenoids With Antioxidant and Antimicrobial Activities.” Waste Management 82: 93–99. 10.1016/j.wasman.2018.10.017. 30509600

[jfds70431-bib-0033] Patidar, M. K. , S. Nighojkar , A. Kumar , and A. Nighojkar . 2018. “Pectinolytic Enzymes‐Solid State Fermentation, Assay Methods and Applications in Fruit Juice Industries: A Review.” 3 Biotech 8, no. 4: 199. 10.1007/s13205-018-1220-4. PMC5866816 29581931

[jfds70431-bib-0034] Pereira, G. V. d. M. , V. d. M. Sampaio , N. Wiele , et al. 2024. “How Yeast Has Transformed the Coffee Market by Creating New Flavors and Aromas Through Modern Post‐Harvest Fermentation Systems.” Trends in Food Science & Technology 151: 104641. 10.1016/j.tifs.2024.104641.

[jfds70431-bib-0035] Pereira, P. V. , D. G. Bravim , R. P. Grillo , et al. 2021. “Microbial Diversity and Chemical Characteristics of *Coffea canephora* Grown in Different Environments and Processed by Dry Method.” World Journal of Microbiology and Biotechnology 37, no. 3: 51. 10.1007/s11274-021-03017-2. 33594606

[jfds70431-bib-0036] Pereira, T. S. , N. N. Batista , L. P. S. Pimenta , et al. 2022. “Self‐Induced Anaerobiosis Coffee Fermentation: Impact on Microbial Communities, Chemical Composition and Sensory Quality of Coffee.” Food Microbiology 103: 103962. 10.1016/j.fm.2021.103962. 35082079

[jfds70431-bib-0037] Prakash, I. , S. R. Shankar , H. P. Sneha , et al. 2022. “Metabolomics and Volatile Fingerprint of Yeast Fermented Robusta Coffee: A Value Added Coffee.” LWT‐Food Science and Technology 154: 112717. 10.1016/j.lwt.2021.112717.

[jfds70431-bib-0038] Ribeiro, L. S. , S. R. Evangelista , M. G. da Cruz Pedrozo Miguel , J. Mullem , C. Ferreira Silva , and R. F. Schwan . 2018. “Microbiological and Chemical‐Sensory Characteristics of Three Coffee Varieties Processed by Wet Fermentation.” Annals of Microbiology 68, no. 10: 705–716. 10.1007/s13213-018-1377-4.

[jfds70431-bib-0039] Schwan, R. F. , N. N. Batista , S. J. Martinez , A. P. P. Bressani , and D. R. Dias . 2022. “Coffee Fermentation: New Approaches to Enhance Quality.” In Coffee Science, 65–98. CRC Press. 10.1201/9781003043133-8.

[jfds70431-bib-0040] Schwan, R. F. , R. M. Cooper , and A. E. Wheals . 1997. “Endopolygalacturonase Secretion by *Kluyveromyces Marxianus* and Other Cocoa Pulp‐Degrading Yeasts.” Enzyme and Microbial Technology 21, no. 4: 234–244. 10.1016/S0141-0229(96)00261-X.

[jfds70431-bib-0041] Shankar, R. S. , H. P. Sneha , I. Prakash , et al. 2022. “Microbial Ecology and Functional Coffee Fermentation Dynamics With *Pichia kudriavzevii* .” Food Microbiology 105: 104012. 10.1016/j.fm.2022.104012. 35473973

[jfds70431-bib-0042] Silva, C. F. , D. M. Vilela , C. d. S. Cordeiro , W. F. Duarte , D. R. Dias , and R. F. Schwan . 2013. “Evaluation of a Potential Starter Culture for Enhance Quality of Coffee Fermentation.” World Journal of Microbiology and Biotechnology 29, no. 2: 235–247. 10.1007/s11274-012-1175-2. 23054699

[jfds70431-bib-0049] dos Santos Silva, M. E. , R. L. de Oliveira , R. M. de Lucena , S. P. da Silva , and T. S. Porto . 2024. “Coffee fermentation as a tool for quality improvement: an integrative review and bibliometric analysis.” International Journal of Food Science and Technology, 59, no. 9: 5912–5925. 10.1111/ijfs.17381.

[jfds70431-bib-0043] Souza, M. L. , F. R. F. Passamani , C. L. da Silva Ávila , L. R. Batista , R. F. Schwan , and C. F. Silva . 2017. “Use of Wild Yeasts as a Biocontrol Agent Against Toxigenic Fungi and OTA Production.” Acta Scientiarum. Agronomy 39, no. 3: 349. 10.4025/actasciagron.v39i3.32659.

[jfds70431-bib-0044] Vilela, D. M. , G. V. M. de Pereira , C. F. Silva , L. R. Batista , and R. F. Schwan . 2010. “Molecular Ecology and Polyphasic Characterization of the Microbiota Associated With Semi‐Dry Processed Coffee (*Coffea arabica* L.).” Food Microbiology 27, no. 8: 1128–1135. 10.1016/j.fm.2010.07.024. 20832694

[jfds70431-bib-0045] Wang, C. , J. Sun , B. Lassabliere , B. Yu , and S. Q. Liu . 2020. “Coffee Flavour Modification Through Controlled Fermentations of Green Coffee Beans by *Saccharomyces Cerevisiae* and *Pichia kluyveri*: Part I. Effects From Individual Yeasts.” Food Research International 136: 109588. 10.1016/j.foodres.2020.109588. 32846616

[jfds70431-bib-0046] Whitaker, J. R. 1990. “Microbial Pectinolytic Enzymes.” In Microbial Enzymes and Biotechnology, 2nd ed., edited by W. M. Fogarty and C. T. Kelly , 133–176. Elsevier Science Ltd.

[jfds70431-bib-0047] Yi, X. , and H. S. Alper . 2022. “Considering Strain Variation and Non‐Type Strains for Yeast Metabolic Engineering Applications.” Life 12, no. 4: 510. 10.3390/life12040510. 35455001 PMC9032683

[jfds70431-bib-0048] Yu, Ai.‐N. , and Ai.‐D. Zhang . 2010. “The Effect of PH on the Formation of Aroma Compounds Produced by Heating a Model System Containing L‐Ascorbic Acid With L‐Threonine/L‐Serine.” Food Chemistry 119, no. 1: 214–219. 10.1016/j.foodchem.2009.06.026.

