# Supplementary material for: Selection and Characterization of Non‐Saccharomyces Yeast Strains for Potential Use in Arabica and Conilon Coffee Fermentations
Source: J Food Sci. 2025 Jul 25;90(7):e70431. doi: 10.1111/1750-3841.70431 (PMC12291027; doi:10.1111/1750-3841.70431)
Supplement: Supplementary file 1 — Supporting Material: jfds70431‐sup‐0001‐SupMat.docx [file JFDS-90-0-s001.docx]

**Supporting Information - Supplementary material**

**Table S1.** Yeast strains isolated from Arabica and Canephora coffee fruits.

| **Yeast strains** | **Coffee species** | **Reference** |
| --- | --- | --- |
| ***Cystofilobasidium ferigula*** |  |  |
| CCMA1619 | *Coffea arabica L*. | Martins et al. (2020) |
| CCMA1621 |  |  |
| CCMA1623 |  |  |
| CCMA1647 |  |  |
| CCMA1636 |  |  |
| CCMA1649 |  |  |
| CCMA1651 |  |  |
| CCMA1654 |  |  |
| CCMA1665 |  |  |
| CCMA1631 |  |  |
| ***Debaryomyces hansenii*** |  |  |
| CCMA0468 | *Coffea arabica L*. | Evangelista et al. (2015) |
| ***Hanseniaspora opuntiae Čadež*** |  |  |
| CCMA1942 | *Coffea arabica L*. | Pereira et al. (2022) |
| ***Hanseniaspora opuntiae Čadež*** |  |  |
| CCMA1733 | *Coffea canephora P.* | Pereira et al. (2021) |
| ***Hanseniaspora uvarum*** |  |  |
| CCMA1639 | *Coffea arabica L*. | Martins et al. (2020)  Pereira et al. (2022) |
| CCMA1892 |  |  |
| CCMA1893 |  |  |
| CCMA1895 |  |  |
| CCMA1944 |  |  |
| CCMA1996 |  |  |
| CCMA 2016 |  |  |
| ***Meyerozyma caribbica*** |  |  |
| CCMA1615 | *Coffea arabica L*. | Martins et al. (2020)  Pereira et al. (2022) |
| CCMA1617 |  |  |
| CCMA1624 |  |  |
| CCMA1634 |  |  |
| CCMA1635 |  |  |
| CCMA1949 |  |  |
| CCMA1950 |  |  |
| CCMA1951 |  |  |
| CCMA1952 |  |  |
| CCMA1953 |  |  |
| CCMA1954 |  |  |
| CCMA1992 |  |  |
| CCMA1993 |  |  |
| CCMA1995 |  |  |
| ***Meyerozyma caribbica*** |  |  |
| CCMA1734 | *Coffea canephora P.* | Pereira et al. (2021) |
| CCMA1735 |  |  |
| CCMA1736 |  |  |
| ***Meyerozyma guilliermondii*** |  |  |
| CCMA1616 | *Coffea arabica L*. | Martins et al. (2020) |
| CCMA1653 |  |  |
| ***Meyerozyma guilliermondii*** |  |  |
| CCMA1737 | *Coffea canephora P.* | Pereira et al. (2021) |
| CCMA1738 |  |  |
| CCMA1739 |  |  |
| CCMA1740 |  |  |
| ***Pichia fermentans*** |  |  |
| CCMA0465 | *Coffea arabica L*. | Evangelista et al. (2015) |
| CCMA0466 |  |  |
| ***Pichia kluyveri*** |  |  |
| CCMA1652 | *Coffea arabica L*. | Martins et al. (2020) |
| CCMA1658 |  |  |
| ***Rhodotorula mucilaginosa*** |  |  |
| CCMA1622 | *Coffea arabica L*. | Martins et al. (2020) |
| CCMA1637 |  |  |
| CCMA1646 |  |  |
| CCMA1662 |  |  |
| CCMA1663 |  |  |
| ***Wickerhamomyces anomalus*** |  |  |
| CCMA1640 | *Coffea arabica L*. | Martins et al. (2020) |
| CCMA1650 |  |  |
| CCMA1659 |  |  |
| CCMA1660 |  |  |

**Table S2.** Identification of volatile compounds (GC-MS peak areas) detected at the end of fermentation in *Coffea arabica* peel and pulp medium.

| **Volatile compounds** | **RI** | **Ions** | **GC-MS peak area** | | | | | | | | | | | |
| --- | --- | --- | --- | --- | --- | --- | --- | --- | --- | --- | --- | --- | --- | --- |
|  |  |  | **Start** | **End of fermentation** | | | | | | | | | | |
|  |  |  |  | **CCMA 1737** | | **CCMA 1993** | | **CCMA 1617** | **CCMA 1992** | **CCMA 1950** | **CCMA 1735** | **CCMA 1658** | **CCMA 1647** | **CCMA 1944** |
| **Acids** |  |  |  |  |  | |  | |  |  |  |  |  |  |
| Acetic acid | 1416 | 43, 45, 60 | 46678 | 22748 | | 25357 | | 46781 | - | 18463.5 | 70156 | - | - | 9986.5 |
| 1,6-Octadien-3-ol, 3,7-  dimethyl- | 1525 | 68, 93, 121 | 49244 | 46124.5 | | 24687 | | 39663.5 | 33584.5 | 37773 | 37083.5 | 22965.5 | 41965 | 31843 |
| Ethyl 9-hexadecenoate | 3015 | 96, 117, 145 | 52874 | 24952 | | 14955 | | 21506 | 3901 | 28929 | 38603 | 34001.5 | 26684 | 48970 |
| Ethyl Oleate | 3645 | 82, 55, 966 | 12066 | 7479 | | - | | - | - | - | 10494.5 | - | - | - |
| (E)-.beta.-Famesene | 3264 | 41, 69, 93 | - | - | | 9651.5 | | - | - | 11502.5 | - | 6298.5 | 5055.5 | 5984.5 |
| 9-Hexadecenoic acid | 2805 | 96, 78, 117 | 10930 | 9948 | | - | | - | - | - | 15829 | - | - | - |
| Nonanoic acid | 2634 | 60, 43, 117 | - | - | | - | | - | - | - | 9947 | - | - | - |
| Total acids |  |  | 171792^a^ | 111251.5^b^ | | 74650.5^c^ | | 107950.5^b^ | 37485.5^d^ | 78204.5^b^ | 182113^a^ | 63265.5^c^ | 73704.5^c^ | 86797.5^b^ |
| **Alcohols** |  |  |  |  | |  | |  |  |  |  |  |  |  |
| 1-Butanol, 3-methyl- | 910 | 55, 70, 88 | - | 427648 | | - | | - | - | - | - | - | - | - |
| 1-Heptanol, 6-methyl- | 1215 | 56, 84, 96 | - | - | | - | | 26206.5 | - | 131871 | - | - | - | - |
| 1-Nonanol | 2555 | 56, 70, 84 | - | 5272 | | - | | - | - | - | 8259.5 | - | - | - |
| 1-Octanol | 1895 | 56, 70, 84 | - | 25992 | | 16957.5 | | 11971 | 8937 | 103519.5 | 23095 | 9809 | 10541 | 10227 |
| Benzyl alcohol | 1950 | 79, 108, 91 | 45307 | 47629 | | 143451 | | 47487.5 | 40411 | 143484 | 56063 | 76682.5 | 86033 | 54337 |
| Cyclohexanemethanol, 4-ethenyl-  .alpha.,.alpha.,4- trimethyl-3-(1-  methylethenyl)- | 2929 | 82, 95, 110 | 9171 | 7458.5 | | 7460.5 | | 8559.5 | 5674 | 8278 | 9421.5 | 6887.5 | 5813 | 3549.5 |
| L-.alpha.-Terpineol | 1730 | 93, 107, 121 | 11837 | - | | - | | 8886 | 6615.5 | - | 11486 | - | - | 7726 |
| Phenylethyl Alcohol | 1991 | 93, 121, 136 | 106462 | 279720.5 | | 4816379.5 | | 182741 | 380067 | 1494865 | 386385.5 | 4429054 | 571270.5 | 2925225.5 |
| (S)-3-Ethyl-4-  Methylpentanol | 1910 | 91, 122, 107 | 46376 | 46830.5 | | - | | 25511.5 | 27056 | 189046 | 50342 | - | - | 43417.5 |
| 1-Dodecanol | 1910 | 55, 70, 85 | 28428 | 51798 | | 9140.5 | | 17658 | 19969.5 | 21580 | 64151 | 9764 | 7421 | 13257.5 |
| E,E-2,13-Octadecadien-  1-ol | 2156 | 55, 70, 83 | - | 116573.5 | | 24554.5 | | 69151 | 55436.5 | 43307 | 132747 | 45783.5 | 35970.5 | 24859.5 |
| 1-Decanol | 2056 | 55, 69, 83 | 13059 | 22274.5 | | - | | 8791 | - | 6683 | 10561.5 | - | - | 13277 |
| Total alcohols |  |  | 260640^b^ | 1031196.5^b^ | | 5017943.5^a^ | | 406963^b^ | 544166.5^b^ | 2142633.5^b^ | 752512^b^ | 4577980.5^a^ | 717049 ^a^ | 3095876.5 ^a^ |
| **Aldehydes** |  |  |  |  | |  | |  |  |  |  |  |  |  |
| Tetradecanal | 2010 | 82, 98, 70 | 21881 | - | | - | | 9863.5 | 7366.5 | - | 13163.5 | - | - | - |
| Heptadecanal | 2156 | 82, 111, 55 | 242226 | 136022.5 | | 24069 | | 165126 | 66944 | 49841.5 | 148895.5 | - | 28267.5 | 20337 |
| Total aldehydes |  |  | 264107^a^ | 136022.5^b^ | | 24069^c^ | | 174989.5^b^ | 74310.5^c^ | 49841.5^c^ | 162059^b^ | - | 28267.5^c^ | 20337^c^ |
| **Ketones** |  |  |  |  | |  | |  |  |  |  |  |  |  |
| 2-Pentadecanone, 6,10,14-trimethyl- | 2518 | 58, 113, 183 | 9321 | 9862.5 | | 2045.5 | | 10101.5 | 4860 | 7492.5 | 11915 | - | - | 4662.5 |
| 4-Hydroxy-3-  methylacetophenone | 1925 | 121, 136, 151 | - | - | | - | | - | - | - | - | - | - | 3394 |
| 7-Acetyl-6-ethyl- 1,1,4,4-  tetramethyltetralin | 3159 | 105, 119, 191 | 1973 | 7310.5 | | 8489 | | 3298 | 8602 | 4798 | 5441 | - | 1284.5 | 5602.5 |
| Total ketones |  |  | 11294^a^ | 17173^a^ | | 10534.5^a^ | | 13399.5^a^ | 13462^a^ | 12290.5^a^ | 17356^a^ | - | 1284.5^b^ | 13659^a^ |
| **Esters** |  |  |  |  | |  | |  |  |  |  |  |  |  |
| 1,2-  Benzenedicarboxylic acid, bis(2- methylpropyl) ester | 3849 | 149, 167, 279 | 107417 | 218310.5 | | 77661.5 | | 140344 | 85525.5 | 130339 | 216008.5 | 49655.5 | 47937.5 | 282245 |
| 2-Ethylhexyl salicylate | 3080 | 120, 178, 207 | 11746 | 16006.5 | | - | | 8182.5 | - | - | 16936 | - | - | - |
| 2-Propenoic acid,  pentadecyl ester | 2075 | 55, 83, 252 | 5226 | 9965.5 | | - | | - | - | - | - | - | - | - |
| 9,12-Octadecadienoic  acid, ethyl ester | 3788 | 67, 88, 264 | 15048 | 5246 | | - | | 4735 | - | - | 9521 | - | - | - |
| Cyclopentanetridecanoic  acid, methyl ester | 2809 | 74, 87, 296 | - | 50188 | | 9842.5 | | 16183 | 8747.5 | 16374 | 60013.5 | 17758 | 10425 | 6915 |
| Dodecanoic acid, ethyl  ester | 1927 | 55, 88, 200 | 168476 | 73523 | | 120194.5 | | 52313.5 | - | 2951926 | 94307.5 | 87720 | 82991.5 | 907630.5 |
| Hexadecanoic acid,  ethyl ester | 2400 | 74, 88, 256 | 90548 | 50419 | | - | | 20710.5 | - | - | 66590.5 | 16414 | - | - |
| Methyl salicylate | 2942 | 120, 138, 152 | 102716 | 49805.5 | | - | | - | - | - | 78804.5 | - | - | - |
| n-Hexadecanoic acid | 4921 | 60, 73, 256 | 50953 | 56754 | | - | | 33084 | - | - | 110462 | - | - | - |
| Octanoic acid | 2100 | 60, 73, 144 | - | - | | - | | - | - | - | - | - | - | - |
| Tetradecanoic acid | 2473 | 60, 73, 228 | 17393 | - | | - | | - | - | - | 31738.5 | - | - | - |
| Tetradecanoic acid,  ethyl ester | 2224 | 60, 88, 256 | 31520 | 13016.5 | | - | | 13174.5 | 10831 | 32926 | 18299.5 | 17150.5 | 13914 | 169067 |
| Hexadecanoic acid,  methyl ester | 2809 | 60, 74, 270 | 49570 | 50419 | | 9687.5 | | 37761 | 8883 | 16175 | 59218.5 | 17758 | 10612 | 8999 |
| 1,2-  Benzenedicarboxylic acid, bis(2- methylpropyl) ester | 2348 | 149, 167, 279 | - | 218310.5 | | 77661.5 | | 140344 | 8552.5 | 130339 | 216008.5 | 49655.5 | 47937.5 | 282245 |
| Total esters |  |  | 650613^b^ | 811964^b^ | | 295047.5^b^ | | 466832^b^ | 122539.5^b^ | 3278079 ^a^ | 977908.5^b^ | 256111.5^b^ | 213817.5^b^ | 1657101.5^b^ |
| **Phenols** |  |  |  |  | |  | |  |  |  |  |  |  |  |
| 2-Methoxy-4- vinylphenol | 2716 | 150, 135, 107 | 4916 | - | | - | | - | - | - | - | - | - | 3500 |
| Phenol, 2,4-bis(1,1-  dimethylethyl)- | 3065 | 191, 205, 220 | 46693 | 75288 | | 81067 | | 16095 | 60371 | 47115 | 61002 | 64509.5 | 66822 | 32675.5 |
| Phenol, 4-ethyl- | 2641 | 107, 122, 94 | - | 46173.5 | | 105314.5 | | 102827 | 19356.5 | 41090 | 127239.5 | - | 4271 | 7278 |
| Phenol, 4-ethyl-2-methoxy- | 2108 | 135, 122, 107 | - | 128201 | | 374709 | | 229886.5 | 53360 | 144901 | 417885.5 | - | 10061.5 | 18766 |
| Total phenols |  |  | 51609^b^ | 249662.5^b^ | | 561090.5^a^ | | 348808.5^b^ | 133087.5^b^ | 233106^b^ | 606127^a^ | 64509.5^b^ | 81154.5^b^ | 62219.5^b^ |

Data are presented as mean. a–b for each column, mean values with different letters are significant at p ≤ 0.05 by Scott–Knott test.

**Table S3.** Identification of volatile compounds (GC-MS peak areas) detected at the end of fermentation in *Coffea canephora* peel and pulp medium.

| **Volatile compounds** | **RI** | **Ions** | **GC-MS peak area** | | | | | |
| --- | --- | --- | --- | --- | --- | --- | --- | --- |
|  |  |  | **Start** | **End of fermentation** | | | | |
|  |  |  |  | **CCMA1895** | **CCMA1663** | **CCMA1652** | **CCMA1950** | **CCMA1944** |
| **Acids** |  |  |  |  |  |  |  |  |
| Acetic acid | 1416 | 43, 45, 60 | 11026 | 416052 | 84507.5 | 26745.5 | 75479 | 27317 |
| Butanoic acid, 3-methyl- | 1624 | 43, 60, 73 | - | - | 6152.5 | 1929 | - | - |
| Dodecanoic acid | 1950 | 60, 73, 117 | 12496 | 329898.5 | 42967 | 100457.5 | 255931 | 51598 |
| n-Decanoic acid | 1991 | 60, 73, 117 | - | 391785.5 | 1433.5 | 8185.5 | 121460.5 | - |
| Octanoic acid | 2100 | 60, 73, 117 | - | 121059.5 | 2528 | 8319 | 55664.5 | 3971.5 |
| 1-Decanol | 1796 | 55, 70, 83 | - | 18324 | 3721 | - | 11250 | 13624 |
| Nonanoic acid | 2634 | 60, 73, 117 | 1223 | 48928 | 2229.5 | 23185.5 | 27257 | 1297 |
| Total acids |  |  | 24745^c^ | 1326047.5^a^ | 143539 ^c^ | 168822 ^c^ | 547042^b^ | 97807.5 ^c^ |
| **Alkanes** |  |  |  |  |  |  |  |  |
| Cyclopropane, 1-heptyl- 2-methyl- | 2056 | 41, 55, 69 | 5753 | 26966.5 | 6662 | 3477.5 | 14381 | 6865.5 |
| Hexadecane | 2099 | 57, 71, 85 | 4709 | 6920.5 | 9666 | 5233 | 11473.5 | 11381 |
| Octacosane | 2132 | 57, 71, 85 | - | 11159 | 6773 | 5797 | - | - |
| Tetradecane | 2283 | 57, 71, 85 | 13729 | - | 13849 | 15827 | - | 21046.5 |
| Total alkanes |  |  | 24191^d^ | 45046^a^ | 36950^b^ | 30334.5^c^ | 25854.5 ^d^ | 39293^b^ |
| **Alcohols** |  |  |  |  |  |  |  |  |
| Phenylethyl Alcohol | 1991 | 93, 121, 136 | 120546 | 794826 | 471126 | 407347.5 | 1700418 | 3595332 |
| 1-Eicosanol | 2318 | 55, 83, 97 | 6009 | 24454.5 | 19138 | 8037 | 35849.5 | 23802 |
| 1-Nonanol | 2555 | 56, 70, 84 | 7551 | 29708 | 7167.5 | 6549.5 | - | 6824 |
| 1-Octanol, 2-butyl- | 2486 | 56, 70, 84 | - | 16140.5 | 7925 | 9159 | 95758.5 | 5642.5 |
| 1-Tetradecanol | 2501 | 55, 83, 97 | 5564 | 21812.5 | 19074 | 7727 | 30678 | 20978.5 |
| 1,6-Octadien-3-ol, 3,7-  dimethyl- | 1540 | 68, 93, 121 | 8222 | 21951.5 | 6360.5 | 4710.5 | 6844 | 8966.5 |
| 2-Dodecanol | 2554 | 55, 70, 83 | 23245 | 140268.5 | 97150 | 63647.5 |  | 95758.5 |
| 2-Nonanol | 2588 | 55, 70, 84 | 95232 | 730514.5 | 105845 | 37992.5 | 225430.5 | 50216.5 |
| Benzyl alcohol | 1750 | 79, 108, 91 | 58942 | 55528.5 | 45061 | 24185.5 | 44339 | 37385 |
| E-11,13-Tetradecadien-  1-ol | 2123 | 55, 69, 83 | - | 7119 | 4328.5 | - | - | - |
| Ethanol, 2-(dodecyloxy)- | 3240 | 57, 71, 85 | 8624 | 28617 | 20656.5 | 26236 | 78688 | 19152 |
| n-Hexadecanoic acid | 2237 | 60, 73, 117 | 41006 | 43105 | 43240.5 | 55691 | 78863.5 | 48377.5 |
| n-Nonadecanol-1 | 1717 | 55, 83, 97 | 2724 | 6762 | 2780 | 5964.5 | 6708.5 | 2863 |
| 1-Dodecanol | 1910 | 55, 70, 85 | - | - | 434109.5 |  | 209820.5 | 327287 |
| Total alcohols |  |  | 377665^f^ | 1920807.5^c^ | 1283962^d^ | 657247.5^e^ | 2513398^b^ | 4242585^a^ |
| **Aldehydes** |  |  |  |  |  |  |  |  |
| Benzaldehyde, 2,4- dimethyl- | 1420 | 105, 77, 51 | 52654 | - | 103851 | - | 22060.5 | - |
| Tetradecanal | 2010 | 82, 98, 70 | 7229 | - | - | 27485 | - | - |
| Total aldehydes |  |  | 59883^b^ | 0 | 103851^a^ | 27485^c^ | 22060.5^c^ | - |
| **Ketones** |  |  |  |  |  |  |  |  |
| 2-Tetradecanone | 1600 | 58, 83, 98 | 2670 | - | 7620 | 5458.5 | 8066 | 9924.5 |
| 2-Undecanone, 6,10-  dimethyl- | 2349 | 43, 57, 70 | 4308 | - | 5729.5 | 3818 | 2823.5 | 6815 |
| 5,9-Undecadien-2-one,  6,10-dimethyl-, (Z)- | 2239 | 67, 81, 95 | 74727 | - | 25628.5 | - | 1022528 | 199908 |
| Total ketones |  |  | 81705^c^ | - | 38978^c^ | 9276.5^c^ | 1033417.5^a^ | 216647.5^b^ |
| **Esters** |  |  |  |  |  |  |  |  |
| 1,2-Benzenedicarboxylic acid, bis(2-methylpropyl) ester | 2348 | 149, 167, 279 | 142023 | 385456 | 147660 | 241533.5 | 209881 | 155194.5 |
| 2-Ethylhexyl salicylate | 3080 | 120, 178, 207 | 17338 | 38062 | 29261.5 | 31315 | 29456.5 | 36139 |
| 2-Propenoic acid, tridecyl ester | 2075 | 55, 83, 252 | - | 10432 | 2826.5 | - | - | - |
| 6-Octadecenoic acid,  methyl ester, (Z)- | 2208 | 55, 69, 83 | - | - | 3166.5 | - | - | - |
| 9-Octadecenoic acid (Z)-  , methyl ester | 2115 | 55, 69, 83 | 3093 | - | - | 2401 | - | 2664.5 |
| 9,12,15-Octadecatrienoic  acid, ethyl ester, (Z,Z,Z)- | 2115 | 55, 69, 83 | 2573 | 2068.5 | 9041.5 | 12926.5 | 5243.5 | 5319 |
| Acetic acid, 2-  phenylethyl ester | 1600 | 104, 77, 105 | 1364522 | 2836266 | 310397.5 | 20763282 | 2324532 | 1423785 |
| Acetic acid,  phenylmethyl ester | 2670 | 105, 77, 91 | 17849 | 53372 | 4810 | 523764 | 24043 | - |
| Benzeneacetic acid, ethyl  ester | 1721 | 91, 105, 77 | - | 88575 | - | 43998 | 20349.5 | 40746.5 |
| Benzoic acid, 2-hydroxy-  , ethyl ester | 2671 | 122, 105, 77 | 98884 | 233694.5 | 88003.5 | 63608 | 69033 | 55212 |
| Decanoic acid, ethyl  ester | 1931 | 88, 101, 143 | 7689 | 968279 | 17680.5 | 86564.5 | 2469347 | 348697 |
| Diamyl phthalate | 2258 | 149, 167, 261 | 14823 | 31280 | 11127.5 | 42322.5 | 37199 | 10951.5 |
| Dodecanoic acid, ethyl  ester | 1927 | 55, 88, 200 | 67695 | 1367428 | 134441 | 269331.5 | 3388525 | 660718.5 |
| Ethyl 9-decenoate | 1725 | 88, 101, 156 | - | - | - | - | 26043 | 27349.5 |
| Ethyl 9-hexadecenoate | 2248 | 88, 101, 156 | 26907 | 124334.5 | 29438.5 | 66779.5 | 78053.5 | 94736 |
| Ethyl Oleate | 2460 | 88, 101, 156 | 6786 | - | 12012 | 14018 | 14752.5 | 12183 |
| Hexadecanoic acid, ethyl ester | 2400 | 74, 88, 256 | 35472 | 42291.5 | 53798.5 | 33668 | 19003.5 | 55519 |
| Hexadecanoic acid, methyl ester | 2809 | 60, 74, 270 | 30704 | 29743 | 29644.5 | 20172 | 10919 | 30925.5 |
| Isopropyl myristate | 2538 | 57, 88, 199 | 4076 | 13045.5 | 15766.5 | 77968.5 | 40049.5 | 29037 |
| Lauryl acetate | 1600 | 70, 83, 129 | - | 16140.5 | 12314 | - | - | 17222 |
| Linoleic acid ethyl ester | 2004 | 88, 101, 263 | 7161 | - | 19150 | 20300.5 | 17924 | 14041.5 |
| Methyl salicylate | 2942 | 120, 138, 152 | 991780 | 1760227 | 908495 | 766049 | 711524.5 | 612345 |
| Oleic Acid | 2004 | 55, 69, 83 | 10314 | 19233.5 | 6138.5 | 11559 | 18453 | 20945 |
| Sulfurous acid, 2-propyl  tetradecyl ester | 2124 | 55, 69, 83 | - | 9962 | 4180 | 9260 | 20267 | 5642.5 |
| Tetradecanoic acid | 2473 | 60, 73, 228 | 8256 | 17093.5 | 11120 | 17192.5 | 28970.5 | 27507.5 |
| Tetradecanoic acid, ethyl  ester | 2224 | 60, 88, 256 | 31049 | 54316.5 | 59838.5 | 22183.5 | 52019 | 218719 |
| Propanoic acid, 2-  methyl-, 1-(1,1- dimethylethyl)-2-methyl- 1,3- propanediyl ester | 1980 | 57, 73, 103 | - | 73625.5 | 44694.5 | - | 9689 | 13413 |
| Total esters |  |  | 2888994^c^ | 8174926^b^ | 1965006.5^c^ | 23140197^a^ | 9625277.5^b^ | 3919013^c^ |
| **Phenols** |  |  |  |  |  |  |  |  |
| 2-Methoxy-4- vinylphenol | 2716 | 150, 135, 107 | 4847 | 3414.5 | 44738.5 | 3553 | 5054 | 9592 |
| Phenol, 2,4-bis(1,1-  dimethylethyl)- | 3065 | 191, 205, 220 | 78526 | 136818.5 | 172881.5 | 127862 | 168998 | 141887.5 |
| Phenol, 4-ethyl- | 2641 | 107, 122, 94 | 4990 | 3509.5 | 16136.5 | 4436.5 | 8084 | 2727 |
| Phenol, 4-ethyl-2-methoxy- | 2108 | 135, 122, 107 | 60036 | 13787.5 | 73265 | 15460 | 36200 | 9773.5 |
| Total phenols |  |  | 148399^a^ | 157530^a^ | 307021.5^a^ | 151311.5^a^ | 218336^a^ | 163980^a^ |

Data are presented as mean. a–b for each column, mean values with different letters are significant at p ≤ 0.05 by Scott–Knott test.
